# Supplementary material for: Thermodynamics and efficiency of an autonomous on-chip Maxwell’s demon
Source: Sci Rep. 2016 Feb 18;6:21126. doi: 10.1038/srep21126 (PMC4758063; doi:10.1038/srep21126)
Supplement: Supplementary Information [file srep21126-s1.pdf]

Supplementary material

Mutual information flow $\dot{I}^Y$ in the fast and slow Demon limits

Mutual information is defined as $I = \sum_{x,y} p_{x,y} \ln[p_{x,y}/(p_x^X p_y^Y)]$, where $p_x^X = \sum_y p_{x,y}$ and $p_y^Y = \sum_x p_{x,y}$ are the marginal distribution functions of the system and the Demon, respectively. In a transition from x, y' to x, y the change of mutual information is given by

$$\Delta I_x^{y'\to y} = \ln \frac{p_{x,y} p_x^X p_{y'}^Y}{p_x^X p_y^Y p_{x,y'}} = \ln \frac{p_{x,y}}{p_{x,y'}}, \tag{i}$$

where we used the symmetry property $p_0^Y = p_1^Y = p_0^X = p_1^X = 1/2$, which results from the fact that the energetically favoured states $(0,1)$ and $(1,0)$ are equally probable to be occupied as are the energetically unfavoured states $(1,1)$ and $(0,0)$.

Fast Demon

By assuming that the Demon is fast enough to thermalize between the transitions in system state x, the conditional probability for state y given that the state of the system is x, $p(y|x)$, is given by

$$p(y|x) = \frac{{}^D\Gamma_x^{y'\to y}}{\gamma^D}, \tag{ii}$$

where $\gamma^D = {}^D\Gamma_x^{y\to y'} + {}^D\Gamma_x^{y'\to y}$. We note that γ^D is independent of x, i.e. ${}^D\Gamma_{x=1}^{y\to y'} + {}^D\Gamma_{x=1}^{y'\to y} = {}^D\Gamma_{x=0}^{y\to y'} + {}^D\Gamma_{x=0}^{y'\to y}$. The joint distribution function $p_{x,y}$ can be then written as

$$p_{x,y} = p_x^X p(y|x) = \frac{1}{2} \times \frac{{}^D\Gamma_x^{y'\to y}}{\gamma^D}. \tag{iii}$$

By inserting the joint distribution function of Eq. (iii) to Eq. (i) we obtain:

$$\Delta I_x^{y'\to y} = \ln \frac{{}^D\Gamma_x^{y'\to y}}{{}^D\Gamma_x^{y\to y'}} \tag{iv}$$

Thus the mutual information flow $\dot{I}^Y$ is given by

$$\dot{I}^Y = \sum_{y\geq y';x} J_x^{y'\to y} \Delta I_x^{y'\to y} = \sum_{y\geq y';x} J_x^{y'\to y} \ln \frac{{}^D\Gamma_x^{y'\to y}}{{}^D\Gamma_x^{y\to y'}} = \beta \dot{Q}_D, \tag{v}$$

where we used the local detailed balance condition for rates ${}^D\Gamma$.

Slow Demon

By assuming that in the slow Demon limit the system thermalizes we obtain

$$p_{x,y} = p_y^Y p(x|y) = \frac{1}{2} \times \frac{W_{x'\to x}^y}{\gamma^X}, \tag{vi}$$

where $\gamma^X = W_{x'\to x}^y + W_{x\to x'}^y$ and rate W was defined in the main text as the sum rate $W = {}^L\Gamma + {}^R\Gamma$ at which the x degree of freedom changes. Similarly as in the fast Demon case, by inserting the joint distribution function of Eq. (vi) to Eq. (i) and using the symmetry relation $p_0^X = p_1^X$ we obtain:

$$\Delta I_x^{y'\to y} = \ln \frac{W_{x'\to x}^y}{W_{x\to x'}^y}. \tag{vii}$$

The mutual information flow is then given by

$$\dot{I}^Y = \sum_{y \geq y';x} J_x^{y' \to y} \Delta I_x^{y' \to y} = \sum_{y \geq y';x} J_x^{y' \to y} \ln \frac{W^y_{x' \to x}}{W^y_{x \to x'}} \tag{viii}$$

Because we operate in the steady state, currents in and out from a state (x, y) must be equal. Thus $J_{x=0}^{y:0\to 1} = J_{x:0\to 1}^{y=1} = J_{x=1}^{y:1\to 0} = J_{x:1\to 0}^{y=0}$. Furthermore, $J_x^{y' \to y} = -J_x^{y \to y'}$ and therefore $J_x^{y' \to y} = -J_{x'}^{y' \to y}$, if $x' \neq x$. By using these relations the mutual information flow of Eq. (viii) is given by

$$\dot{I}^Y = -\sum_{x \geq x';y} J^y_{x' \to x} \ln \frac{W^y_{x' \to x}}{W^y_{x \to x'}} = -\dot{\sigma}^X \tag{ix}$$

Derivation of the efficiency of the measurement-feedback cycle ϵ_T

Since the energy cost for tunnelings events in the system are related as ${}^{L(R)}E^{y=0}_{x:0\to 1} = {}^{R(L)}E^{y=1}_{x:1\to 0}$, as can seen from Eq. (3) of the main text, the rates W satisfy a relation $W^{y=0}_{x:0\to 1} = W^{y=1}_{x:1\to 0}$ and $W^{y=1}_{x:0\to 1} = W^{y=0}_{x:1\to 0}$. By using these relations and the fact that $J^{y=0}_{x:0\to 1} = -J^{y=1}_{x:0\to 1}$, we obtain

$$\dot{\sigma}^X = \sum_{x \geq x';y} J^y_{x' \to x} \ln \frac{W^y_{x' \to x}}{W^y_{x \to x'}} = J^{y=0}_{x:0\to 1} \ln \frac{W^0_{0\to 1}}{W^0_{1\to 0}} + J^{y=1}_{x:0\to 1} \ln \frac{W^1_{0\to 1}}{W^1_{1\to 0}} = 2J^{y=0}_{x:0\to 1} \ln \frac{W^0_{0\to 1}}{W^0_{1\to 0}} \tag{x}$$

The entropy production rate in the Demon is given by

$$\beta \dot{Q}_D = \sum_{y \geq y';x} J_x^{y' \to y} \ln \frac{{}^D\Gamma_x^{y' \to y}}{{}^D\Gamma_x^{y \to y'}} = J^{y:0\to 1}_{x=0} \ln \frac{{}^D\Gamma^{y:0\to 1}_{x=0}}{{}^D\Gamma^{y:1\to 0}_{x=0}} + J^{y:0\to 1}_{x=1} \ln \frac{{}^D\Gamma^{y:0\to 1}_{x=1}}{{}^D\Gamma^{y:1\to 0}_{x=1}} = J^{y:0\to 1}_{x=0} \beta\kappa, \tag{xi}$$

where we used the fact that $J^{y:0\to 1}_{x=0} = -J^{y:0\to 1}_{x=1}$ and local detailed balance condition of rate ${}^D\Gamma$. Thus, combining Eqs. (x) and (xi) and using $J^{y:0\to 1}_{x=0} = -J^{y=0}_{x:0\to 1}$ we obtain

$$\frac{-\sigma^X}{\beta \dot{Q}_D} = \frac{2}{\beta\kappa} \sigma^X_r, \tag{xii}$$

where $\sigma^X_r = \ln [W^0_{0\to 1}/W^0_{1\to 0}]$.

Derivation of Eqs. (15) and (16) of the main text

In a system tunneling event from (x', y) to (x, y) the coarse grained entropy production is given by

$$\sigma^X = \ln \frac{{}^L\Gamma^y_{x' \to x} + {}^R\Gamma^y_{x' \to x}}{{}^L\Gamma^y_{x \to x'} + {}^R\Gamma^y_{x \to x'}} \tag{xiii}$$

The conditional probability to tunnel over the left/right junction is given by

$$P_{L/R} = \frac{{}^{L/R}\Gamma^y_{x' \to x}}{{}^{L/R}\Gamma^y_{x' \to x} + {}^{R/L}\Gamma^y_{x' \to x}}. \tag{xiv}$$

Thus

$$P_L e^{-\beta Q_L} + P_L e^{-\beta Q_R} = P_L \exp[-\ln \frac{{}^L\Gamma^{xx'}_y}{{}^L\Gamma^{x'x}_y}] + P_R \exp[-\ln \frac{{}^R\Gamma^{xx'}_y}{{}^R\Gamma^{x'x}_y}] = e^{-\sigma^X}. \tag{xv}$$

The average $\langle e^{-\beta Q_S} \rangle$ over a process of fixed amount time is given by

$$\langle e^{-\beta Q_S} \rangle = \langle e^{-\beta Q_L} \xi^L + e^{-\beta Q_R} \xi^R \rangle = \langle e^{-\sigma^X} \rangle, \tag{xvi}$$

where $\xi^{L/R}$ is the indicator function giving $\xi^{L/R} = 1$ if the tunneling is over the left/right junction and 0 otherwise. The equation above can be written as

$$\langle e^{-(\beta Q_S - \sigma^X)} \rangle = \langle e^{-S_{cg}} \rangle = 1, \tag{xvii}$$

where S_{cg} is the coarse graining cost.
